# Study of in-vitro metabolism of selected antibiotic drugs in human liver microsomes by liquid chromatography coupled with tandem mass spectrometry

**DOI:** 10.1007/s00216-016-9929-6

**Published:** 2016-10-04

**Authors:** Malgorzata Szultka-Mlynska, Boguslaw Buszewski

**Affiliations:** 1 Department of Environmental Chemistry and Bioanalytics, Faculty of Chemistry, Nicolaus Copernicus University, Gagarin 7, 87-100 Torun, Poland; 2 Center for Modern Interdisciplinary Technologies, Nicolaus Copernicus University, Wilenska 4, 87-100 Torun, Poland

**Keywords:** Antibiotic drugs, Human liver microsomes, In-vitro, Metabolism, Mass spectrometry

## Abstract

High performance liquid chromatography coupled with triple-quadrupole mass spectrometry was applied in the determination of in vitro metabolism products of selected antibiotic drugs (cefotaxime, ciprofloxacin, fluconazole, gentamicin, clindamycin, linezolid, and metronidazole). The analytes were separated on a reversed phase C18 column, with acetonitrile and 0.1 % aqueous formic acid as the mobile phase. Tandem mass spectrometry with positive electrospray ionization was used to facilitate the structural characterization of the potential metabolites. Metabolism studies on human liver microsomes were performed via cytochromes P450 (phase I) and via NADPH/UDP-glucuronosyltransferase (phase II) mediated reactions. LC-MS/MS experiments allowed potential metabolite peaks, including sum formulae suggestions, to be identified; high resolution MS/MS experiments led to the identification of various oxidative and reductive modifications of target compounds in phase I biotransformation, and conjugation products with glucuronic acid in phase II reactions. A total of 11 potential metabolites and their proposed structures were characterized during the incubation of human liver microsomes by comparing their retention times and spectral patterns with those of the parent drug. Dehydrogenation and reactions of side chains such as hydroxylation and hydrolysis of ester bonds constituted the major metabolic pathways. Finally, LC-MS/MS spectrometry was revealed to be a suitable analytical tool to procure a feasible analytical base for the envisioned in vivo experiments.

**Electronic supplementary material:**

The online version of this article (doi:10.1007/s00216-016-9929-6) contains supplementary material, which is available to authorized users.

**Graphical Abstract:**
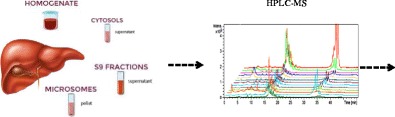
Workflow overview of in vitro drug metabolism studies.

## Introduction

In recent years, a significant interest regarding bacterial infections could be observed. Moreover, resistance to commercially available antimicrobial agents by pathogenic microorganisms has been increasing at an alarming rate and has become a serious global problem for healthcare. On the other hand, therapeutic drug monitoring (TDM) referring to the individualization of dosage is an important aspect of interdisciplinary approach towards such studies. Therefore, TDM will be more valuable, which will make it possible to personalize and optimize therapeutic practices and healthcare system. Moreover, in view of the diverse and unique pharmacokinetic profile of drugs in patients undergoing treatment for bacterial infections, there is a need to use TDM in an attempt to optimize the exposure to antibiotics, improve clinical outcome, and limit the alarming occurrences of antibiotic resistance in bacteria [1–3]. Antibiotics are a class of antimicrobials, a larger group that also includes anti-viral, anti-fungal, and anti-parasitic drugs. They are among the most frequently prescribed medications in modern medicine. There is a wide range of antibiotics, and the choice of what to administer to a patient depends on which kind of infection it is and which types of antibiotics are known to be effective against it as different classes of antibiotics combat bacterial infections in different ways. They are widely prescribed; however, the fact that microorganisms are developing resistance to these drugs means that their efficacy may be lost and care should be taken to avoid unnecessary administration [4, 5].

Typical in-vitro methods in metabolic studies include microsomal approaches using human liver microsomes (HLMs). Liver is the major organ involved in the biotransformation of various endogenous compounds and drugs. Moreover, liver cells show a high level of cytochrome P450 activity and often are the main site of metabolic transformation. Liquid chromatography-tandem mass spectrometry (LC-MS/MS) has become a powerful and reliable analytical approach for metabolite quantitation because of its high sensitivity, low consumption, and high speed of analysis [6–16].

**Fig. 1:**
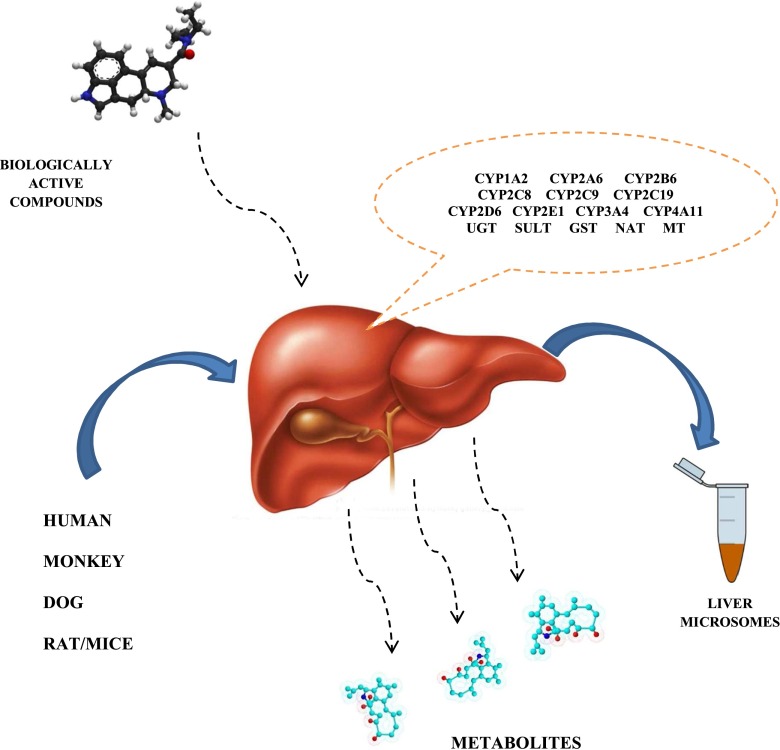
Scheme of isolation of subcellular microsomal fractions applied in evaluation the in vitro metabolism of biologically active compounds

Because the liver is the major organ for drug metabolism, increased throughput screening assays have been developed to determine the metabolic stability of drugs. Two experimental systems—liver microsomes and intact hepatocytes—are currently widely applied (Fig. 1). Moreover, combining electrochemistry with mass spectrometry creates a powerful analytical tool for metabolite studies and helps overcome many of the laborious tasks by isolating the metabolites formed in vivo (blood, plasma, urine) or in vitro (microsomes, artificial vein system (HLM) [15].

As there are no publications regarding the interaction of the researched analytes (cefotaxime, ciprofloxacin, fluconazole, gentamicin, clindamycin, linezolid, and metronidazole) with human liver enzymes of microsomal fraction (HLM), a study was undertaken to elucidate this issue. To the best of our knowledge, no detailed information regarding the structure of their potential metabolites via in vitro studies has been published. In this study, we applied LC/MS to identify phase I and phase II metabolites of the studied biologically active compounds detected after in vitro incubation of the researched. Transformations were investigated with HLM and in the presence of NADPH/UDPGA-P450 oxidoreductase. Additionally, obtained results were compared to in vivo studies with the use of biological samples from patients. The results obtained will allow predicting metabolic pathways of target compounds in patients differing in the level of cytochrome P450 isoenzymes and, in turn, can probably be helpful in designing personalized antibacterial treatments including these drugs.

## Materials and methods

### Chemicals and materials

Cefotaxime (CEF), ciprofloxacin (CIP), fluconazole (FLU), gentamicin (GEN), clindamycin (KLI), linezolid (LIN), metronidazole (MET), and gemifloxacin (GEMI) (internal standard, IS) were obtained from Sigma-Aldrich (Schnelldorf, Mittelfranken, Germany). Glucose-6-phosphate, β-NADP+, glucose-6-phosphate dehydrogenase, and uridine 5′-diphosphoglucuronic acid triammonium salt (UDPGA) were obtained from Sigma-Aldrich (Schnelldorf, Mittelfranken, Germany). Other chemicals of analytical quality (acetonitrile, formic acid) were purchased from Merck (Darmstadt, Germany). Microsomes derived from human liver cells (HLMs) at the concentration of 10 mg/vial were used in enzymatic studies. The microsomal fractions were stored at –80̊ °C and thawed immediately before each experiment. Ultrapure water was used for all analyses. Other chemicals were all analytical reagents. The water was obtained by means of Milli-Q RG apparatus by Millipore (Millipore Intertech, Bedford, MA, USA) in our laboratory. Drug-free urine was kindly provided by Nicolaus Copernicus University, Collegium Medicum, Torun (Poland) with Bioethical Commission permission (no. 639/2010).

### Phase I in vitro incubation

The antibiotic drug incubation (concentration 1–25 μm) with HLMs was performed in phosphate buffer (pH 7.4) at 37 °C. Then, the incubated samples were shaken for a specified time at 120 rpm. The optimization of conditions for carrying out the process included the incubation time, the concentration of HLM, the concentration of analytes, and the reaction buffer consisting of an enzymatic reaction cofactor NADP/NADPH, glucose-6-phosphate dehydrogenase, and magnesium ions. The blank samples were of the same composition as the test samples; however, they did not contain any of the studied biologically active compounds. The process was terminated with 50 μL of ice-cold ACN. Thereafter, samples were immediately prepared and analyzed with the use of HPLC-UV/MS. All samples were prepared in triplicate. Glucuronidation was studied by incubation of the model drug with HLMs. The test and blank samples were prepared analogously to phase I metabolism samples, the exception being that 10 μL of PB contained the UDPGA co-factor, which mediated the relevant reaction. All samples were incubated and treated as described above and as also described in detail in reference [16].

### Cancer cell line

Human Caucasian colon adenocarcinoma cell line (Caco-2) was obtained from ECACC (European Collection of Cell Cultures, UK). Cells were cultured in Dulbecco’s modified Eagle medium – high glucose 4.5 g/L Sigma-Aldrich (Schnelldorf, Mittelfranken, Germany), supplemented with 20 % (v/v) inactivated fetal bovine serum (PAA Laboratories GmbH, Pasching, Austria) and 1 % (v/v) nonessential amino acids (Biological Industries, Kibbutz Beit-Haemek, Israel). Additionally, gentamycin from Sigma-Aldrich (Schnelldorf, Germany) was added to the culture media at the concentration 0.04 mg/mL. The study used a special culture dish in the form of a closed, two-chamber container. Between the upper and the lower chamber, there was a horizontal porous membrane on which a culture of epithelial cells grew. Both the upper (apical) and the lower (basal) chamber were filled with growth medium. The pH of the growth medium on the apical side was 6.5, whereas on the basal side it was 7.4, which simulates in vivo conditions. Cells were cultured at 37 °C in humidified atmosphere of 5 % CO_2_. Caco-2 cells were plated in 96-well plates (Thermo Fisher Scientific, Nunc, Denmark) at the density of 6 × 10^4^ cells per cm^2^ and grown for 72 h. Then the medium was aspirated for 12 or 24 h. Finally, gentamicin and its potential metabolites were analyzed after filtration from the culture medium.

### Chromatography and mass spectrometry

The presented procedure is based on the procedure described in reference [16]. An overview of the tandem mass spectrometry (MS/MS) settings is listed in Table 1.

**Table 1:** MS-MS conditions used for determination of selected antibiotic drugs in human liver microsomes

Analyte	Abbreviation	Ion mode; [M + H]^+^	Retention time (min) (mean, SD)	Parent ion, *m/z*	Quantifier ion[*Q1*], *m/z*	Qualifier ion[*Q2*], *m/z*	Collision energy (eV); *Q1*, *Q2*	Drying gas temperature (˚C)	Fragmentor	Capillary voltage (V)	multiple reaction monitoring ratio (mean, CV%)
Cefotaxime	CEF	Positive	5.28 ± 0.10	456	396	342	22, 36	290	105	3500	1.23
Ciprofloxacin	CIP	Positive	5.15 ± 0.15	332	314	288	27, 24	350	195	4000	1.13
Fluconazole	FLU	Positive	5.06 ± 0.10	307	289	220	29, 35	320	135	4000	1.14
Gentamicin	GEN	Positive	7.15 ± 0.10	478	322	-	29	290	165	4500	1.59
Clindamycin	KLI	Positive	6.87 ± 0.15	425	162	377	31, 28	290	165	4500	2.81
Linezolid	LIN	Positive	4.35 ± 0.15	338	322	296	30, 35	320	135	4000	1.12
Metronidazole	MET	Positive	4.58 ± 0.10	172	128	-	25	320	105	3500	1.44

### Calibration and validation

A validation of the developed method was performed according to the appropriate approaches described in detail in references [16–18].

### Sample preparation – urine assay

The patient urine samples contained slight amounts of urine proteins; therefore, a protein pretreatment was necessary. Three mL of acetonitrile was added to 1.5 mL of the urine sample to remove the protein. The mixture was shaken for 10 min and then centrifuged at 7500×*g* force to obtain 4 mL of the supernatant. The supernatant was diluted with a mobile phase and then was filtered through a 0.22-mm microporous membrane. The filtrated samples were used for HPLC measurements.

## Results

### In vitro incubation of selected antibiotic drugs with human liver microsomes

**Fig. 2:**
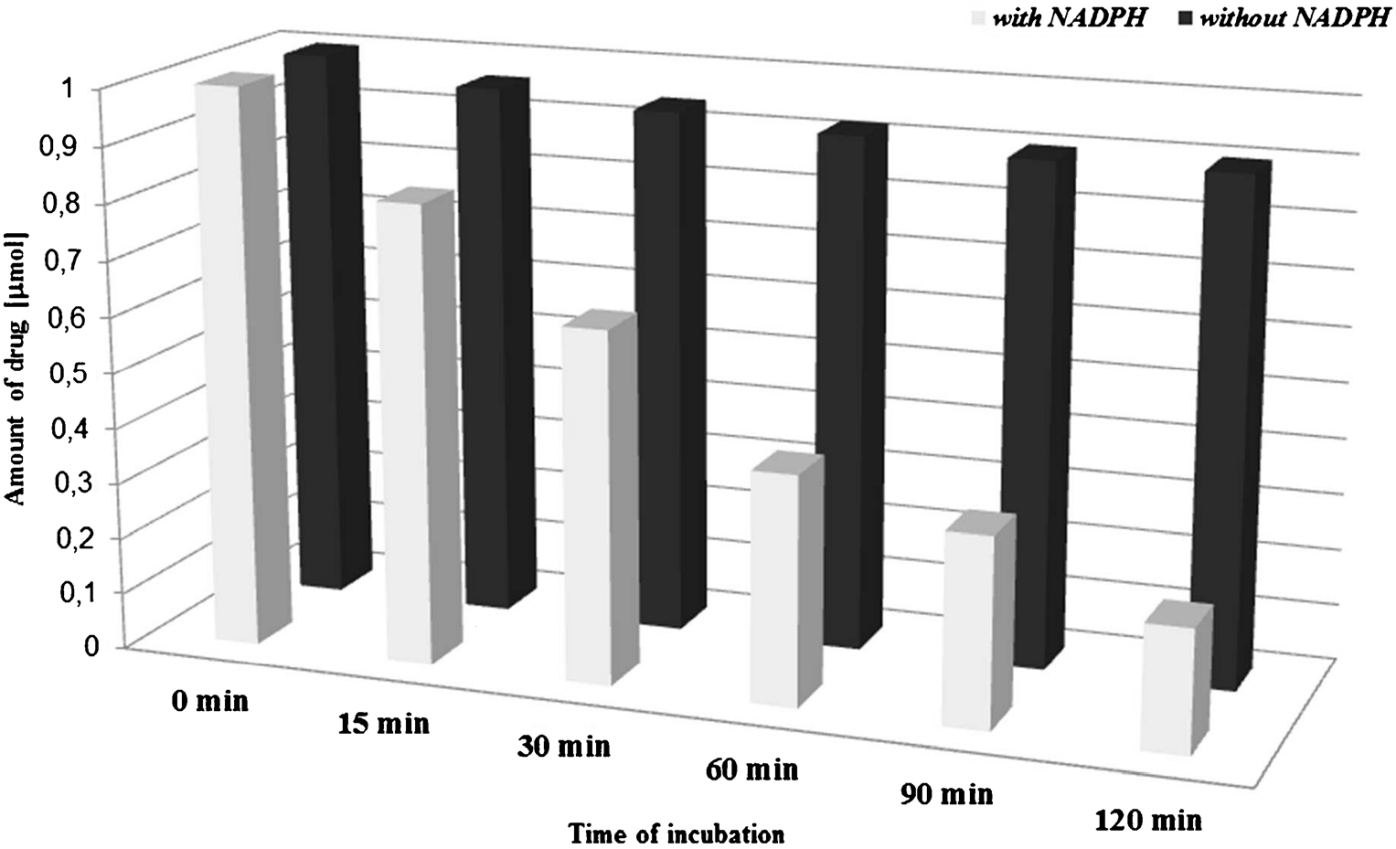
The influence of the presence of enzymatic reaction cofactor-NADPH for antibiotic drug (FLU) conversion. Pre-incubation of human liver microsomes at 37 °C before the NADPH addition (black diagram) and with pre-incubation of HLMs together with NADPH (gray diagram)

Human liver microsomes were applied during performed investigation. Seven antibiotic drugs different in physicochemical structure were included in the incubation tests, namely ciprofloxacin (CIP), cefotaxime (CEF), fluconazole (FLU), gentamicin (GEN), clindamycin (KLI), linezolid (LIN), and metronidazole (MET). To select the right conditions for the experiment, initially the studied compounds were incubated in the conditions appropriate for carrying out the process, which included several parameters such as the incubation time (0–120 min), concentration of HLM (0.2–1.6 mg/mL), concentration of the selected antibiotic drug (10–25 μM), and reaction buffer consisting of an enzyme reaction cofactor NADPH/UDPGA (1.5–30 mM), glucose-6-phosphate (G-6-P) (0.5–15 mg/mL), dehydrogenase (0.8–24 U/mL), and magnesium ions (1.5–7.5 mM). The incubation with enzymes of microsomal fraction was performed in phosphate buffer (pH 7.4) at 37 °C. Heating of liver microsomal fraction enzymes at 37 °C for a set period of time in the absence of NADPH had no influence on the amount of the substance in the reaction mixture. In other words, the collected results suggest that the studied antibacterial medicines were susceptible to enzymatic transformations in the presence of metabolic biotransformation cofactor, and the degree to which they reacted depended both on the concentration of NADPH and on the incubation length. The results are illustrated in Fig. 2, with fluconazole serving as reference. As the graph displays, the concentration of the substrate showed linear decrease as the reaction progressed.

On the basis of the results obtained, the following parameters were applied for further studies: 120 min of incubation time; 0.4 mg/mL of HLM, 10 μM of selected antibiotic drug, 5 mM of NADPH/UDPGA, 10 mg/mL G-6-P, 16 U/mL of dehydrogenase, and 5 mM of MgCl_2_.

### Identification of antibiotic drugs metabolites

**Fig. 3:**
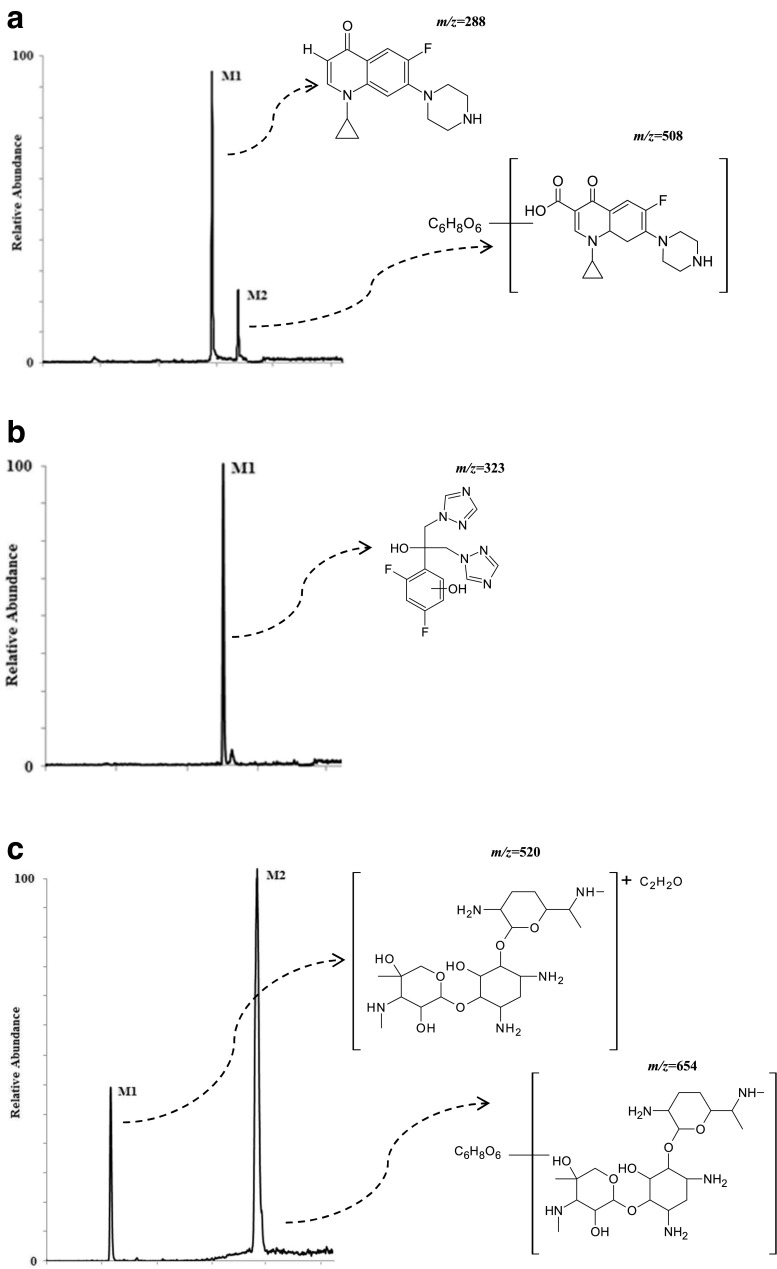

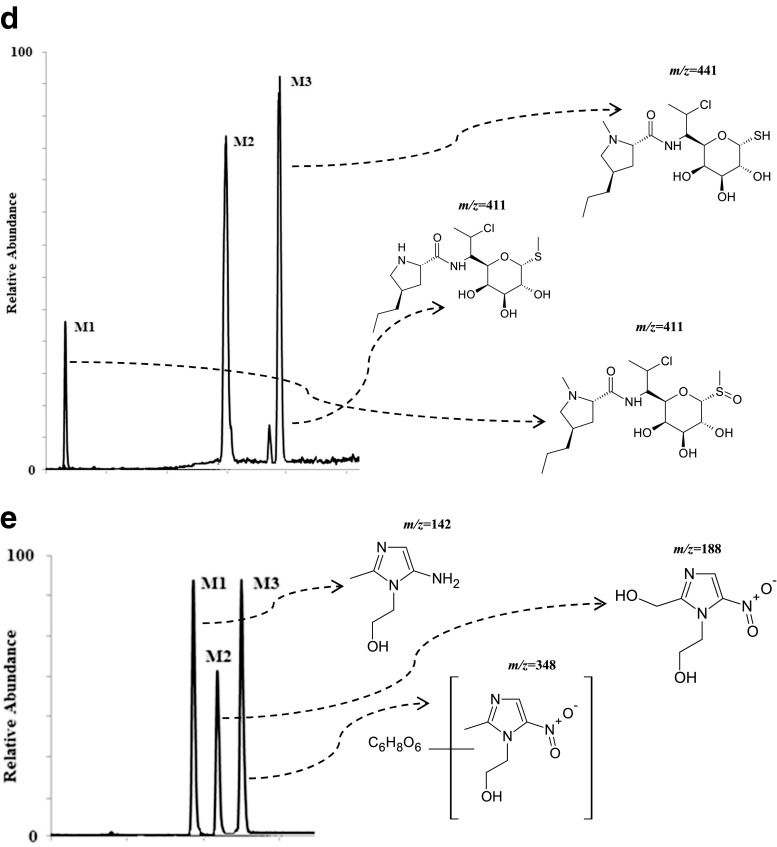
Representative extracted ion chromatogram of potential metabolites produced following the incubation with human liver microsomes for (**a**) CIP; (**b**) FLU; (**c**) GEN; (**d**) KLI, and (**e**) MET

The analysis of the presented MS chromatograms reveals that most of the studied antibiotic drugs undergo enzymatic transformations very easily, and the yield depends on the presence and concentration of NADPH and on incubation time. Biotransformation of the target compounds leads to creation of one, two, or three main products (Fig. 3). Based on the gathered results, a conclusion was drawn that there were metabolites of the phase I of biotransformation reaction, and metabolites of phase II. The potential metabolites were assigned the numbers M1–M3 referring to their retention times.

Additionally, the experiments on two-stage MS/MS fragmentation for a selected protonated molecule (a potential metabolite) confirmed the validity of their postulated chemical structures. This is in agreement with the general knowledge that this group of cytochrome P450 isoenzymes participates in metabolism of numerous drugs. However, for certain products the peaks were of low intensity, whereas a large fraction of the substrate had reacted and concentration of the products decreased with time of the reaction; finding an explanation of this phenomenon turned out to be a problem. One may speculate that the appearing reactive metabolites quickly bind to the surface of microsomal proteins present in the mixture at high concentration. In case of CIP, M1 metabolite was observed at *m/z* 288, which points to decarboxylation of the side chain. Additionally, in the presence of the other enzymatic reaction cofactor, UDPGA, the creation of potential M2 metabolite was observed ([M + H]^+^ for *m/z* 508). It corresponded to conjugation of the basic drug with glucuronic acid. As a result, the MS/MS spectrum revealed a loss of a defined neutral fragment – glucuronic acid (Δ-176 Da) and a signal from the secondary ion for *m/z* 208, which appears during fragmentation of a precursor ion for the resulting compound. For FLU, metabolite M1 was observed at *m/z* 323, indicating hydroxylation of the aromatic moiety. In case of GEN, metabolite M1 corresponded to *m/z* 520, indicating acetylation. Moreover, in the presence of UDPGA, the creation of potential M2 metabolite was observed ([M + H]^+^ for *m/z* 654), corresponding to conjugation of the basic drug with glucuronic acid. For KLI, the obtained metabolites at *m/z* 411 and 441 are probably associated with the S-dealkylation or N-dealkylation and S-oxidation, respectively. In case of MET, metabolites M1 (*m/z* 142) and M2 (*m/z* 188) correspond to reduction of nitro groups and oxidation of aliphatic chains, respectively. Furthermore, in the presence of UDPGA, the creation of potential M3 metabolite was observed ([M + H]^+^ for *m/z* 348), and corresponding conjugation of the basic drug with glucuronic acid. Table 2 presents the retention times, chemical structures, *m/z* ratio, composition, and proposed biochemical reaction for each of the chosen antibacterial drugs. Moreover, the protonated molecular ions, retention times, and main MS2 product ions of target compound metabolites are presented in Table 2.

**Table 2:**
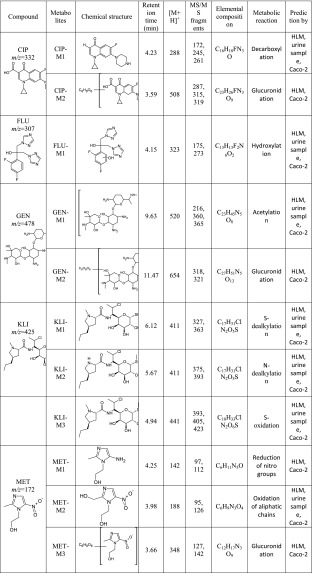
The retention times, mass characteristics and elemental composition of the [M + H]^+^ of studied antibiotic drugs and their potential metabolites

### In vitro studies with the use of Caco-2 cell line

**Fig. 4:**
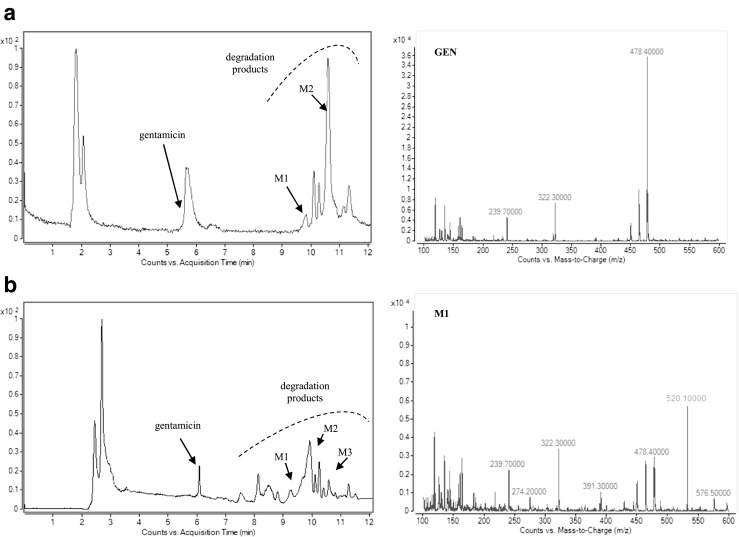

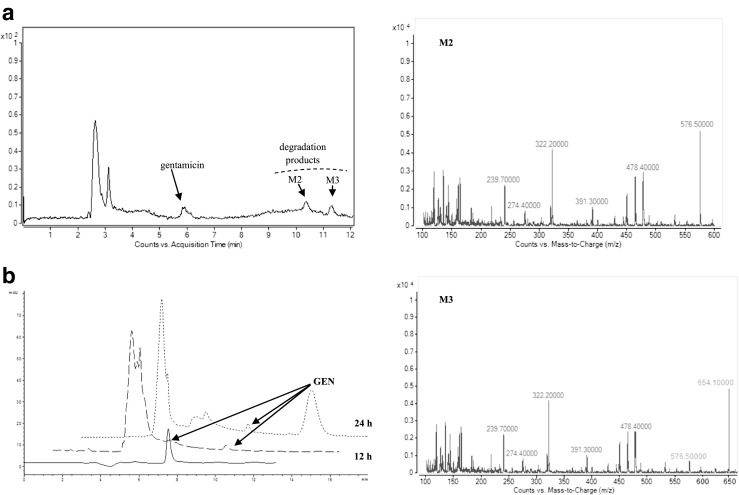
Total ion chromatograms and mass spectra of gentamicin and their potential metabolites after incubation with Caco-2 cell line: (**a**) after 6 h, (**b**) after 12 h, (**c**) after 24 h, (**d**) UV chromatogram after 12 h and 24 h. Mass spectrometric detection was carried out in ESI(+) mode

With the knowledge of the directions that metabolic transformations of gentamycin as a model compound in the enzymatic system can take, it was decided that the next step in the research would be to study its biotransformation in another area of its expected therapeutic (bactericidal) action (i.e., in cancer cells of the Caco-2 line). An appropriate chromatographic image of the contents of methanol extracts isolated from Caco-2 cells was obtained for cell concentration of 2 × 10^3^, gentamycin concentration of 100 μg/mL, and incubation time of 6, 12, and 24 h. Based on the collected results, it was concluded that Caco-2 cells at this stage of growth show metabolic activity. The studies of timed exposure of Caco-2 culture to the studied substance proved that intestinal epithelial cells can activate processes leading to biotransformation of the drug. Studies on metabolic biotransformation of gentamycin in the presence of enzymes of Caco-2 cell line reveal that at least three main metabolites are created. The MS spectra of the peaks of M1, M2, and M3 products contain mainly mass ions with respective values of 520, 576, and 654. The growth of the molecular weight of the substrate (gentamycin), by 42, 96, and 176 units, respectively, signifies that these entities are products of phase II metabolic reactions. It was also observed that the reactions occur independently of each other. Chromatographic analysis revealed the presence of three GEN metabolites (Fig. 4). It can be concluded that the drug penetrated epithelial cells to a slight degree and underwent metabolic transformations. After 24 h of reacting, the characteristic signals from potential metabolites were disappearing, which could be caused by their instability. The peak of M2 metabolite is particularly interesting as although its intensity decreases with incubation time, it is still present in the mixture after 24 h. The appearance of a new metabolite, which had not been observed during the transformation of the chosen analyte in the presence of human microsomes, underlines the existing differences concerning how easily they undergo metabolic transformations in the presence of microsomal proteins found in various models for in vitro studies. This knowledge can be helpful to prognose the influence of the studied antibiotic drugs on the activity of metabolizing enzymes and in planning a targeted therapy. However, such interactions can be different in vivo. The results derived from this approach were compared with HLM experiments as well as in vivo experiments by analyzing real urine samples from patients after antibiotic drugs have been administered. Table 2 may be consulted in order to obtain a quick overview of all metabolites discussed in this study, with specific reference to their chemical structure, mass data, and fragmentation patterns.

### Method validation

After the sample preparation and HPLC/MS conditions were defined, a validation was performed to assess the performance of the proposed methods. Intra-day accuracy and precision of target compounds in human liver microsomes are shown in Table S2 (see Electronic Supplementary Material, ESM). Accuracy and precision results are presented in Table S3 (see ESM). Stability of the target compounds in biological samples was tested at three concentration levels (1, 7, and 15 μg/mL) under different conditions. The results of stability tests for biological samples are presented in Table S4 (see ESM). Additionally, possible matrix effect on the signals for target compounds was evaluated by studying the difference between the mass spectrometric signal for studied antibiotic drugs in standard solutions and the signal for these compounds in a biological matrix (e.g., HLM). Less than 15 % matrix effect was observed for studied antibiotic drugs.

## Discussion

The HPLC parameters for the HPLC-UV-MS/MS method were selected after screening a number of columns and solvent systems with different isocratic profiles. The conditions selected in the developed procedure (see Experimental section) gave excellent determination with run time lower than 15 min. To select the LC-MS/MS parameters, a standard amount of 1 μg/mL of target compound was injected into the HPLC column in full scan in positive and negative modes. ESI positive ion mode provided the best response, where the protonated molecular ion [M + H]^+^ relevant for the target antibiotic drug was monitored very well on the MS chromatograms. Some parameters of ESI source such as capillary voltage (3500–4500 V), nebulizer gas pressure (30–40 psi), drying gas flow (6–8 L/min), drying gas temperature (290–350 °C), and fragmentor voltage (75–255 V) were optimized to obtain most intense detector response (see ESM Fig. S1). Each parameter was optimized separately. Mass spectrometric detection was performed using electrospray and the mass spectrometer was operated in multiple reaction monitoring mode. The described analytical method was prepared for simultaneous qualitative analysis of target antibiotic drugs and their potential metabolites. Each of the antibiotic drugs was also quantified.

Moreover, an attempt was made to determine chemical structures of the products that are the outcome of metabolic biotransformation of the tested drugs. For this purpose, mass spectra were recorded for each studied analyte (substrate) and potential metabolite (product) with HPLC-ESI/MS technique. There is a vast number of individual components in human liver microsomal fraction enzymes that can elute during the analysis at the same time as the target substance. To solve this problem, the study utilized Mass Hunter Metabolite ID. software. With this tool, the signals coming from a given analyte were compared with reference samples with regard to their retention times, MS spectra, and areas. To fulfill requirements for confirmatory methods, two transitions were monitored for each analyte. For each analyte, one transition was chosen for quantification, and the second was used for confirmation. MS/MS was used to determine whether the fragmentation of target compounds is specific and makes it possible to identify them in the next stages of research (e.g., during the determination and identification of potential metabolites). The most intense signal on the full scan spectra was chosen as the parent ion. The first stage of research was focused on the selection of collision energy, which was tested in the range of 20 to 40 eV, increasing energy of 1–5 eV. The obtained results showed that MS/MS will be useful in further studies as a tool for qualitative analysis because of the vast possibility to confirm the structure of the metabolites produced on the basis of the product ion spectra. Detailed MS-MS conditions are listed in Table 3. Ion groups [M + H]^+^ for the proposed metabolites were initially determined and identified in full scan mode. With preselected mass spectrometer analysis conditions, CEF was identified as [M + H]^+^ for *m/z* 456, CIP as [M + H]^+^ for *m/z* 332, FLU as [M + H]^+^ for *m/z* 307, GEN as [M + H]^+^ for *m/z* 478, KLI as [M + H]^+^ for *m/z* 425, LIN as [M + H]^+^ for *m/z* 338, and MET as [M + H]^+^ for *m/z* 172. Multistage fragmentation of target compounds was carried out in order to gather data for interpreting MS spectra of potential metabolites of the selected drugs.

**Table 3:**
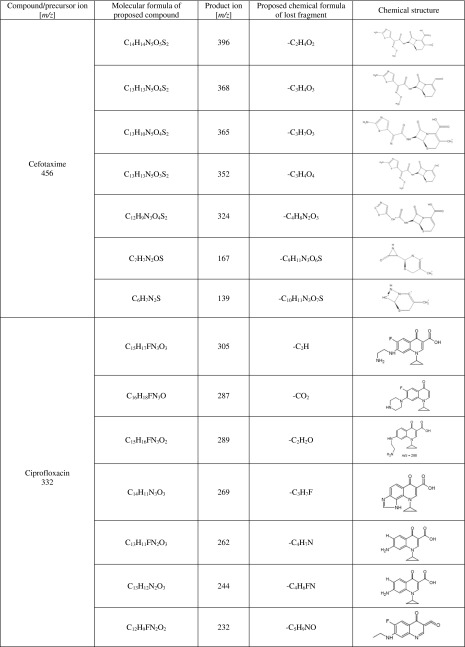

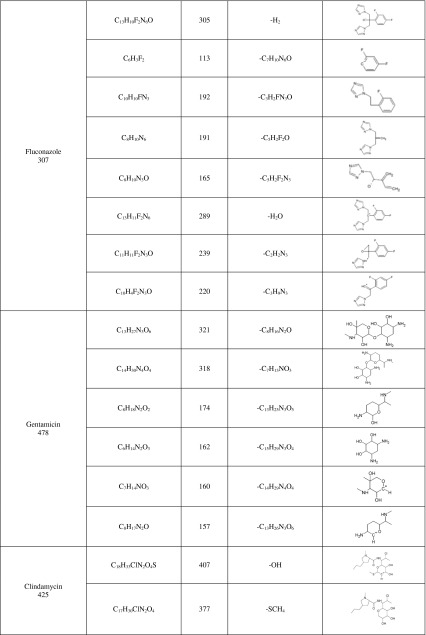

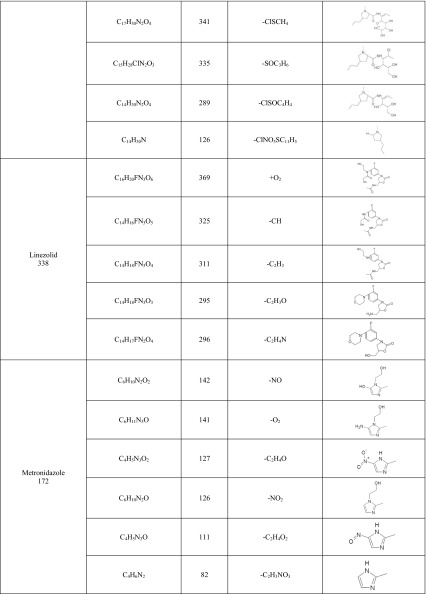
Ions observed by ESI-MS/MS

Studies on the metabolism of target compounds were carried out with human liver microsomes. The reactions were performed under a wide range of incubation conditions and were monitored by HPLC-UV/MS. The rates of substrate transformations were calculated as the ratio of the substrate HPLC peak height after a given time of incubation to the initial peak height of the substrate. On the basis of the results obtained for cefotaxime and linezolid, one can conclude that they are not in the substrate spectrum of the action of the studied enzymes. Besides cytochrome P450, other important elements of microsomal fraction of liver cells include flavin monooxigenase (FMO) and glucuronosyltransferase (UDP). Research on linezolid and cefotaxime, which contain a methoxyl group instead of a hydroxyl one, aimed at establishing potential substrate properties in the presence of another metabolizing enzyme. Thus, in research on determining the enzyme groups present in microsomal vesicles of liver cells that can participate in transformation of the studied analytes, the next step was to check how easily LIN and CEF undergo transformations in the presence of phase II metabolic enzymes – glucuronosyltransferases (i.e., UGT). Considering the above described reactivity of these compounds observed after incubation with a mixture of microsomal enzymes, the outcome of the tests run for linezolid and cefotaxime was surprising. As a consequence, it led to formulating several possible working hypotheses. One can assume that LIN and CEF, owing to their chemical structure and pharmacological properties, do not constitute a substrate for any of the tested isoenzymes of cytochrome P450. It is thus possible that other isoenzymes belonging to cytochrome P450 play a part in the metabolism of the studied drugs. Therefore it is likely that metabolic processes occurring outside liver cells can be important for biotransformation of both substances. Considering this, metabolism in liver cells seems not to be the only way these substances are transformed. As metabolic transformations of the selected drugs in the presence of human liver microsomal fraction enzymes were observed, it was concluded that these substances showed varied ability to transform. The collected results allowed us to determine that human microsomes are an appropriate material for research as well as for making a prognosis concerning the metabolic transformations that the tested analytes can be undergoing in a patient’s organism. Enzymatic transformations of the chosen substances in the presence of microsomal enzymes were conducted in conditions selected with the aim to activate an appropriate antibacterial drug. Among the multiple microsomal fraction enzymes of liver cells, the ones most involved in biotransformations of foreign substances are isoenzymes of cytochrome P450; ca. 95 % of currently used medicines are metabolized by this group of enzymes [19]. Table S1 (see ESM) presents the human liver microsomal fraction enzymes used in this study, together with their enzymatic activity.

Unchanged in relation to the substrates, the character of the UV-Vis spectra (data not presented) of biotransformation products of specific medicines supports the assumption that in their particles, changes in the side chain can be expected. In the case of ciprofloxacin, the changes in the structure of the drug molecule occur in the carboxyl group. In the case of fluconazole, the changes in molecule structure resulting from enzymatic biotransformation concern the benzyl group. During the tests, no phase II product was observed for fluconazole and clindamycin. Probably these drugs do not undergo phase II biotransformation reactions in the presence of glucuronosyl transferase enzymes located in the microsomal fraction of human liver. To confirm the noticeable participation of UGT in creating phase II biotransformation products for the other medicines, β-glucuronidase was added to the reaction mixture. Activity of this enzyme results in hydrolysis of glucuronides [20]. During incubation in the presence of bacterial enzyme, it was noticed that the peaks of products of metabolic glucuronidation were gradually disappearing. This observation is also a proof that in a living organism there is a relation between phase I and phase II of metabolism. Besides, the presence and the appropriate level of NADPH-dependent cytochrome P450 reductase in liver cells is a necessary condition for proper physiological actions of its isoenzymes. Interpretation of the collected mass spectra of the peaks of the obtained metabolites allows us to conclude that the other studied antibacterial drugs easily undergo enzymatic transformations in the presence of selected co-substrates. The *m/z* values of their mass ions can correspond to chemical structures of the products in which, after the first phase of metabolic transformations, there occurred conjugation of the given medicine with the glucuronic acid radical (Δ-176 Da) through the oxygen atom in the drug particle. Presence of such metabolites also confirms the supposition that a product of phase I of metabolic biotransformation is further transformed during phase II, in this case by glucuronidation.

When a given drug was incubated without cofactor presence, no signals coming from other substances were observed during the analysis. The testing also included the susceptibility of the chosen analytes to transformations when exposed to phase II metabolic enzymes present in the microsomal vesicles of liver cells, i.e., glucuronosyltransferases (UGT). They are responsible for metabolizing a wide range of clinically important compounds. Glucuronosyltransferases are the main phase II biotransformation enzymes, playing a significant role in the detoxification process of a number of endo- and exogenous substances in the organisms of humans and other vertebrates. Catalyzed by the abovementioned group of enzymes, reactions of glucuronidation of – often dangerous – toxic substances and drug metabolites lead to an increase of their polarity, which facilitates excretion with urine or bile. During glucuronidation reaction, glucuronic acid in its active form of UDPGA conjugates with a functional group of the drug, usually hydroxyl, carboxyl, amino, or thiol, which results in creation of (usually less toxic) O-, N-, or S-glucuronides. Multiple variations of the UGT family enzymes are present in endoplasmic reticulum and cellular membranes. Located mainly in the liver, they also play a significant role in metabolic transformations occurring outside liver cells—in the alimentary canal and kidneys [21].

The proposed study used a cell line of human colon cancer which, owing to the presence of phase I and II metabolic enzymes, is commonly used in studying metabolic transformations of many drugs and xenobiotics [22]. A research on metabolic transformation of the selected drug first required determination of suitable reaction conditions by selecting the amount of cells, substance concentration level, and time of its incubation with the cells. Popularity of epithelial cell lines in research on biotransformation is primarily due to the fact that application of this relatively simple experimental model quickly yields information that can be generalized. The Caco-2 cell line was isolated from a colon tumor found in a 72-y-old Caucasian man. During culturing it undergoes spontaneous differentiation, and owing to its enterocyte-like structure, it is used as a model line in in-vitro cultures. Caco-2 cells produce such enzymes as disaccharidase, peptidase, CYP 450 isoenzymes, glutathione-S transferase, sulfotransferase, and glucuronidase, as well as transport proteins produced by absorptive epithelial cells, which participate in transport of saccharides, amino acids, peptides, and vitamins. It also produces p-glycoprotein and multidrug resistance proteins, involved in transport and metabolism of therapeutic agents. Furthermore, on the apical side of their surface, the cells of this line produce small amounts of intestinal mucus [22]. Epithelial cells are cultured at 37 °C in special incubators with atmospheric content regulated so that the amount of CO_2_ equals 5 %, which imitates the concentration of this gas in blood. Proper humidity protects the culture from desiccating.

## Conclusions

The proposed analytical procedure can be useful in pharmacokinetics, in experiments on incubating microsomes derived from liver samples collected from patients treated with antibiotics. Besides, although the total level of liver microsomes P450 does not vary significantly, genetic polymorphism and susceptibility to induction of particular isoenzymes result in individual differences in drug metabolism. Ultimately, for each medicine the characteristic phase I and phase II metabolic biotransformation reactions were identified so as to facilitate identification of new substances for personalized antibiotic therapy in further advanced research. All the postulated phase I metabolic reactions are typical for processes catalyzed by isoenzymes of the cytochrome P450 group. It may be concluded that the human liver microsomes with their enzymatic activity may be very useful in the study of the metabolism of antibiotic drugs.

## Electronic supplementary material

Below is the link to the electronic supplementary material.

ESM 1 (PDF 143 kb)
