# Supplementary material for: Study of in-vitro metabolism of selected antibiotic drugs in human liver microsomes by liquid chromatography coupled with tandem mass spectrometry
Source: Anal Bioanal Chem. 2016 Oct 4;408(29):8273–87. doi: 10.1007/s00216-016-9929-6 (PMC5116318; doi:10.1007/s00216-016-9929-6)
Supplement: Supplementary file 1 — (PDF 143 kb) [file 216_2016_9929_MOESM1_ESM.pdf]

Analytical and Bioanalytical Chemistry

Electronic Supplementary Material

Study of in-vitro metabolism of selected antibiotic drugs in human liver microsomes by liquid chromatography coupled with tandem mass spectrometry

Malgorzata Szultka-Mlynska, Boguslaw Buszewski

Table S1 Characteristic of izoenzymes used during the study

Enzyme	Enzymatic activity [pmol/(mg×min)]
Total P450	250 pmole/mg
Cyt. b_5	460 pmole/mg
CYP1A2	820
CYP2A6	1200
CYP2B6	28
CYP2C8	190
CYP2C9	3000
CYP2C19	53
CYP2D6	99
CYP2E1	1900
CYP3A4	4900
CYP4A11	3500
FMO	680
UGT1A1	920
UGT1A4	890
UGT1A9	2400

Table S2 Intra-day accuracy and precision of target compounds in human liver microsomes

Compound	Theoretical concentration [mg/ml]	Observed concentration [mean±SD]	Accuracy [%]	RSD
CEF (n=10)	1	1.1±0.3	110.0	6.83
	3	3.3±0.1	110.0	1.66
	5	5.3±0.7	106.0	6.25
	7	6.9±0.4	98.5	0.24
	10	9.5±0.4	95.0	3.10
	15	15.2±0.3	101.3	3.26
	20	20.1±0.4	100.5	2.44
	30	28.9±0.1	96.3	2.90
	50	47.3±0.6	94.6	2.76
CIP (n=10)	1	1.1±0.3	110.0	7.03
	3	2.9±0.1	96.6	1.70
	5	5.1±0.7	102.0	6.43
	7	6.7±0.4	95.7	0.24
	10	9.8±0.4	98.0	3.19
	15	15.1±0.3	100.6	3.35
	20	20.2±0.4	101.0	2.51
	30	29.3±0.1	97.6	2.98
	50	48.2±0.6	96.4	2.84
FLU (n=10)	1	1.1±0.3	110.0	6.69
	3	3.1±0.1	103.3	1.62
	5	5.2±0.7	104.0	6.12
	7	7.1±0.4	101.4	0.23
	10	9.9±0.4	99.0	3.03
	15	15.2±0.3	101.3	3.19
	20	19.9±0.4	99.5	2.39
	30	30.1±0.1	100.3	2.84
	50	49.4±0.6	98.8	2.70
GEN (n=10)	1	1.1±0.3	110.0	6.48
	3	3.1±0.1	103.3	1.57
	5	5.1±0.7	102.0	5.93
	7	7.2±0.4	102.8	0.22
	10	9.8±0.4	98.0	2.94
	15	15.1±0.3	100.6	3.09
	20	20.3±0.4	101.5	2.31
	30	29.5±0.1	98.3	2.75
	50	48.6±0.6	97.2	2.62
KLI (n=10)	1	0.9±0.3	90.0	6.89
	3	3.2±0.1	106.6	1.67
	5	5.1±0.7	102.0	6.31
	7	7.1±0.4	101.4	0.24
	10	9.7±0.4	97.0	3.13
	15	15.1±0.3	100.6	3.29
	20	20.3±0.4	101.5	2.46
	30	29.7±0.1	99.0	2.92
	50	49.2±0.6	98.4	2.78
LIN (n=10)	1	0.9±0.3	90.0	7.01
	3	2.8±0.1	93.3	1.70
	5	5.1±0.7	102.0	6.40
	7	7.3±0.4	104.2	0.24
	10	9.9±0.4	99.0	3.17
	15	15.1±0.3	100.6	3.34
	20	20.4±0.4	102.0	2.50
	30	30.2±0.1	100.6	2.97
	50	50.2±0.6	100.4	2.82
MET	1	1.1±0.3	110.0	6.97

(n=10)	3	3.1±0.1	103.3	1.69
	5	5.1±0.7	102.0	6.38
	7	7.2±0.4	102.8	0.24
	10	9.8±0.4	98.0	3.16
	15	14.8±0.3	98.6	3.32
	20	19.9±0.4	99.5	2.49
	30	29.3±0.1	97.6	2.96
	50	49.5±0.6	99.0	2.81

Table S3 Accuracy and precision results

Compound	Nominal concentation (µg/ml)		Intraday (n=4)		Interday (n=3)	
			%Bias	%CV	%Bias	%CV
Cefotaxime	LQC	1	-2.5	10.8	-10.7	12.6
	MQC	20	-0.5	11.7	-5.0	7.9
	HQC	50	-2.8	6.1	-6.9	5.6
Ciprofloxacin	LQC	1	-1.1	12.4	-9.6	11.8
	MQC	20	-0.9	10.3	-5.8	6.5
	HQC	50	-3.1	7.2	-3.5	3.9
Fluconazole	LQC	1	-0.8	10.8	-10.3	11.5
	MQC	20	-0.9	5.9	-7.4	7.5
	HQC	50	-3.5	2.9	-3.9	4.9
Gentamicin	LQC	1	-1.2	9.6	-9.4	12.3
	MQC	20	-0.8	6.8	-6.4	10.6
	HQC	50	-4.4	2.7	-2.9	8.5
Clindamycin	LQC	1	-1.3	12.1	-9.4	12.2
	MQC	20	-0.9	9.6	-8.3	8.9
	HQC	50	-4.2	3.8	-3.7	5.4
Linezolid	LQC	1	-1.2	10.6	-10.2	11.4
	MQC	20	-1.1	5.9	-5.9	9.6
	HQC	50	-3.9	3.7	-3.8	6.8
Metronidazole	LQC	1	-1.1	9.5	-9.5	10.5
	MQC	20	-0.9	5.8	-8.3	9.7
	HQC	50	-4.4	3.4	-4.2	3.5

Table S4 The stability of target compounds in human liver microsomes under different conditions

Antibiotic drug	Amount added	Stability			
		Short-term stability [12 h/20℃]	Long-term stability [30 days/-20℃]	Freeze-thaw stability [-20℃ to room temparature]	Post-preparative stability [24 h/10℃]
		Amount found [mean±SD]			
CEF	1 μg/ml	1.2±0.82	1.3±1.11	1.2±0.48	1.1±0.36
	7 μg/ml	6.9±0.39	7.2±1.29	7.3±2.11	7.1±0.94
	15 μg/ml	15.1±1.21	15.2±4.21	14.9±1.34	15.3±0.87
CIP	1 μg/ml	1.1±0.68	1.2±1.13	1.1±0.49	1.2±0.39
	7μg/ml	7.1±0.42	7.1±1.49	7.2±2.18	7.4±0.99
	15 μg/ml	15.3±1.18	15.1±3.21	15.2±1.35	15.1±0.57
FLU	1 μg/ml	1.1±0.72	1.3±1.17	1.1±0.98	1.2±0.76
	7 μg/ml	7.3±0.69	7.2±1.25	7.3±2.61	7.1±0.95
	15 μg/ml	15.2±1.51	15.1±4.71	15.4±1.36	15.1±1.17
GEN	1 μg/ml	1.1±0.89	1.1±1.15	1.1±0.58	1.2±0.85
	7 μg/ml	7.2±0.69	7.1±1.69	7.1±3.15	7.2±2.58
	15 μg/ml	15.1±1.69	15.2±2.38	14.8±1.35	15.1±0.59
KLI	1 μg/ml	1.2±0.69	1.1±1.19	1.1±0.98	1.2±0.74
	7 μg/ml	7.1±0.45	7.2±1.35	7.1±2.72	7.2±0.97
	15 μg/ml	15.2±1.89	15.1±3.28	15.1±1.59	15.2±1.49
LIN	1 μg/ml	1.1±3.85	1.1±1.19	1.2±1.38	1.2±0.58
	7 μg/ml	6.9±0.59	7.1±2.28	7.2±4.18	7.1±2.38
	15 μg/ml	15.2±1.39	15.1±4.28	14.9±1.28	15.1±0.84
MET	1 μg/ml	1.1±2.88	1.2±1.15	1.1±2.92	1.2±1.38
	7 μg/ml	6.9±0.39	7.2±1.29	7.3±2.15	7.3±0.29
	15 μg/ml	15.1±1.21	15.2±4.21	14.9±1.3	15.3±0.87

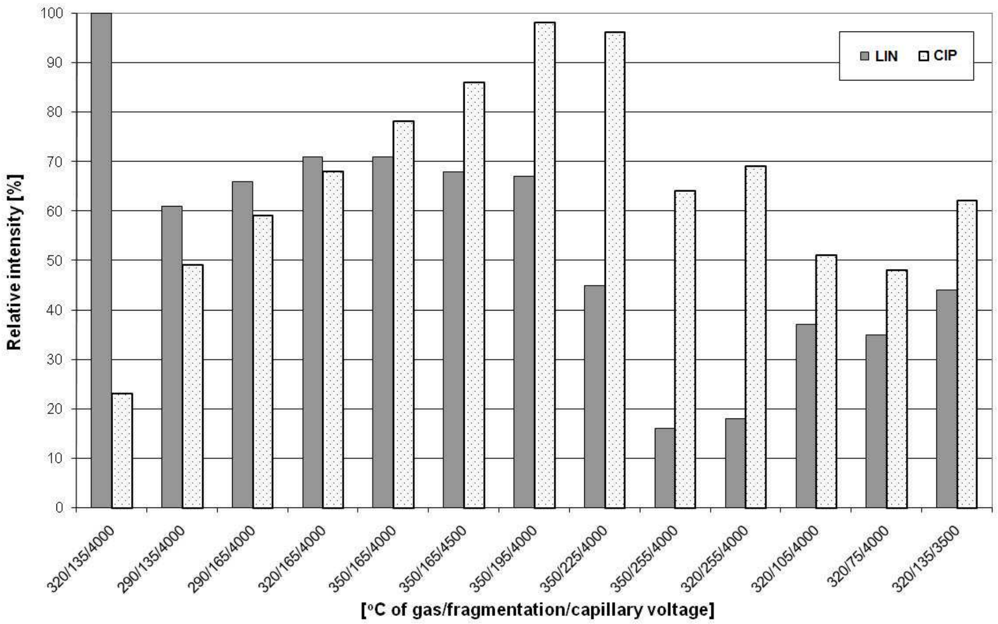

Fig. S1 Comparison of the relative intensity at *m/z*=338 $[M+H]^+$ for LIN and *m/z*=332 $[M+H]^+$ for CIP using different values of parameters: drying gas temperature (DGT), fragmentation (F), capillary voltage (CV)
